# Navigating complex decision in a new setting; perspectives of Arabic-speaking migrant men in Sweden on family planning decision making

**DOI:** 10.1371/journal.pone.0325935

**Published:** 2025-06-11

**Authors:** Mazen Baroudi, Elin C. Larsson, Helena Kilander

**Affiliations:** 1 Department of Epidemiology and Global Health, Umeå University, Sweden; 2 Department of Women’s and Children’s Health, Karolinska Institutet, and the WHO collaborating centre, Karolinska University Hospital, Stockholm, Sweden; 3 Department of Global Public Health, Karolinska Institutet, Stockholm, Sweden; 4 Jönköping Academy for Improvement of Health and Welfare, School of Health and Welfare, Jönköping University, Jönköping, Sweden; Faculty of Social Sciences, Vytautas Magnus University, LITHUANIA

## Abstract

**Background:**

Male partners’ involvement in family planning can be crucial for women’s contraceptive choices. Limited research exists on migrant men’s experiences and perceptions of family planning and associated decisions. The study aims to investigate experiences and perceptions of family planning use and decision-making among Arabic-speaking men living in Sweden.

**Methods:**

This qualitative study utilized reflexive thematic analysis of eighteen in-depth interviews. The participants aged 22–43 years, had relocated to Sweden within the past decade, and represented various countries of origin (e.g., Syria, Iraq, Palestine) with most participants having 13 or more years of education.

**Results:**

We identified an overarching theme, “*Navigating the complex family planning decision-making in a new setting*” supported by four sub-theme; 1) “*The new living conditions influencing family planning decision-making*” described how financial (in)stability, childcare responsibilities, personal development goals, and the impact of changing circumstances shaped family planning choices; 2) “*Social norms affecting family planning decision-making*” emphasized the diversity of gender roles and norms within Arabic-speaking communities, with an emphasis on joint decisions and shared responsibility. Instances of controlling behaviour and the role of beliefs and extended family members were also noted; 3) “*Challenges in accessing contraceptive services free of discrimination*” underscored the need for and importance of access to comprehensive information and culturally sensitive services in shaping family planning decisions; 4) “*Conflicting considerations when deciding on contraceptive method*” addressed factors such as familiarity with the methods, perceived effectiveness and safety, fear of side-effects, and taboos associated with the methods.

**Conclusions:**

The findings highlight the diverse factors influencing family planning decisions. The study underscores men’s willingness to be active partners for family planning and highlights the need for improved information provision and services to empower informed decision-making.

## Introduction

Men’s involvement in sexual and reproductive health (SRH) has a positive impact not only on their own SRH but also on gender equality and women’s sexual and reproductive health and rights (SRHR) [1]. To strengthen women’s SRHR, it is key to ensure women’s ability to make choices in family planning and to access contraception [2]. Family planning refers to a conscious effort by individuals or a couple to limit or space the number of children they have through the use of contraceptive methods or treatment of involuntary infertility [3]. Traditionally, family planning has focused on women, despite the fact that men are highly involved in reproduction [4]. It is still unclear whether counselling including both men and women increases contraceptive use compared to counselling for women alone [5]. However, studies from different settings show the value of involving men in family planning because men’s support is considered to indirectly affect women’s opportunities to plan pregnancies including contraceptive decision making [4,6,7].

Migrant women across countries of all income groups report lower access to contraceptive services, accompanied by higher rates of unintended pregnancies and abortions [8–10]. Ensuring access to effective contraception is central when seeking to decrease inequities that migrants face in SRHR as it prevents unintended pregnancies and enables birth spacing [2]. Although migrant men can play a supportive role, there is still limited understanding regarding migrant men’s experiences of family planning use and contraceptive decision making [11]. A study conducted with Somali migrant men in Sweden showed their awareness of the benefits of family planning but also fear of using modern contraceptives, highlighting their need for counselling [12]. Healthcare providers (HCPs) usually express concerns regarding the potential impact of migrant men’s involvement on women’s reproductive autonomy [13]. Interestingly, HCPs usually fail to recognize the supportive role that migrant men can assume and the importance that migrant women attribute to their partners’ involvement [13].

The extent of men’s involvement and inclusion in family planning and contraceptive decision-making in Sweden remains unclear. Prior research has highlighted limited access, organizational structural barriers, and undefined roles concerning men’s access to SRH services [11,12,14]. This is especially relevant among migrant men in Sweden because they report higher instances of sexual risk-taking and lower condom use [15,16]. Moreover, studies underscored the lack of access to information and SRHR education among Arabic-speaking migrant men [17]. Although a heterogeneous group, Arabic-speaking migrant men constitute the largest migrant group in Sweden, migrating to Sweden from various countries such as Syria, Iraq, and Palestine [18].

Overall, there is a knowledge gap regarding how services should be designed to reduce the inequities migrants face in accessing SRHR [4,6] including how to ease Arabic-speaking men’s support in women’s contraceptive choices.

## Materials and methods

### Study aim

In order to improve contraceptive services and counselling for Arabic speakers migrated to Sweden, we performed this study aiming to investigate Arabic-speaking men’s experiences and perceptions of family planning use and decision making.

### Study setting

This qualitative study is part of two projects. The first project, “Improve -it”, is a quality improvement project aiming at developing postpartum contraception services for migrants. The project includes qualitative interviews with migrant women and men and a codesign of intervention together with migrant women and midwives in maternal health clinics utilizing a cluster randomized controlled trial design [19]. The second project, Migrants’ sexual and reproductive health and rights – MSRHR, is a qualitative interview study examining the perceptions and experiences of Arabic-speaking migrant men regarding SRH information and services in Sweden [17].

In Sweden. Midwives are the main providers of contraceptive services [20]. Contraceptives are subsidized up to 26 years of age [21] and interpreting services are offered at no cost during healthcare appointments to migrants who do not speak Swedish, as per their request (Förvaltningslag § 13 (2017:900), [Administration Act], 2017).

Migrant men represent a substantial share of the population in Sweden, with around 20% of all men in the country born abroad. Of these, about 25% come from the so-called Arabic world [18].

### Study design

A qualitative design and methodology using reflexive thematic analysis was chosen to study the phenomena of family planning and decision making, and to enable deeper understanding of participants´ perceptions and experiences. The results are reported in accordance with the Consolidated Criteria for Reporting Qualitative Research (COREQ) checklist.

The preliminary plan for this article was to conduct focus group discussions with Arabic-speaking men similar to the study design conducted with Arabic-speaking women within the IMPROVE-it project, but due to the difficulty in recruitment, we opted to conduct individual interviews. The difficulty in recruitment could be partly attributed to the sensitive nature of the topic, particularly within the context of Sweden’s current anti-migration political climate. Additionally, Arabic and Muslim migrants may have concerns about the potential detention of their children under the “Act with Special Provisions on the Care of Young People (LVU)”. This law allows authorities to take children into custody if their safety or well-being is at risk, a concern that may influence their willingness to participate in discussions about family decision-making [22,23]. Following a pragmatic approach and due to time and resource constraints, we stopped data collection after conducting seven interviews.

After the preliminary analysis of the seven interviews conducted under the Improve-it project between 01-10-2022 and 31-01-2023, we decided to include excerpts from transcripts of 11 out of 13 interviews from the MSRHR project which was conducted between 01-10-2020 and 31-03-2021 by the first author. The 11 interviews were included because they included men’s perspective about contraceptive use and decisions of family planning [17]. These interviews included questions about the needs, experiences and perceptions of SRH information and services among Arabic speaking men in Sweden including questions about contraceptive use that were not analyzed in the previous paper [17].

In both projects described above, we employed the following recruitment methods: visits to Swedish language schools, Facebook announcements at groups and pages oriented to SRHR or Arabic migrants in Sweden and snowball technique. This approach allowed us to engage a broader range of potential participants and capture a diverse set of experiences during the interviews.

Under the *improve-it* project, the interviewees were fathers, ranged in age from 31 to 43 years and had relocated to Sweden within the past decade. The participants represented different countries of origin and educational background. Under MSRHR project, the interviews were conducted with men aged 22–37 years who have (3 participants) or do not have children (8 participants). Table 1 presents the socio-demographic characteristics of the 18 participants in the two projects.

**Table 1:** Participants’ (N = 18) characteristics of the Improve-it and the MSRHR projects.

		Improve -it (n = 7)	MSRHR (n = 11)
Age	18–24	0	3
	25–34	2	5
	35+	5	3
Years in Sweden	1 to 2 years	2	1
	3 to 5 years	2	2
	5 + years	3	8
Have children	Yes	7	3
	No	0	8
Child born in Sweden	Yes	5	3
	No	2	0
Country of origin	Syria	3	4
	Iraq	2	2
	Palestine	1	3
	Other	1	2
Years in education	Up to 9 years	1	1
	10 to 12 years	1	2
	13 + years	5	8

This article is, therefore, based on interviews from two projects (Improve-it and MSRHR) which were analyzed based on the same research question.

The integration of the two datasets allowed us to include Arabic-speaking men living in different parts of Sweden and from different age groups, civil status and different perceptions and experiences regarding family planning decision process. Following this pragmatic approach, we believe that we addressed our research question effectively and are satisfied with the structure of the developed themes.

### Data collection

Migrants in this study refers to people who were born outside Sweden and moved to Sweden without consideration to the reason of migration or the length of the stay.

Interested individuals were initially provided with both oral and written information about the study, including a consent form. Once a participant agreed to take part in the interview, a mutually convenient time was scheduled for a separate occasion.

The interview guide for the *Improve-it* project included questions about the participants’ knowledge, attitude, and experiences of contraception and contraceptive counselling in Sweden and how can these services be improved (for more details see Supporting Information, S1 File). The excerpts of interviews from the *MSRHR project* were related to the perceptions and attitudes of Arabic-speaking migrants’ men in relation to contraceptive use and their roles in it (the interview guide of the *MSRHR project* is published here [17]).

The first author, a native Arabic-speaking medical doctor, conducted all the interviews in Arabic. The interviews were conducted through video or audio-conferencing tools (Zoom and WhatsApp) and were recorded using a separate external dictaphone. All interviews were conducted online due to the concurrent Covid-19 pandemic during the *MSRHR* project and due to the participant’s preference in the *Improve-it* project. The semi-structured interviews had an average duration of 39 minutes, ranging from 22 to 57 minutes. During the first interviews, we piloted the interview guides with the participants and made necessary adaptations to the questions and probing techniques. Throughout the interviews, the interviewer employed probing and follow-up questions to delve deeper into the participants’ answers and opinions. The interviewer also provided summaries of the participants’ responses, asking for their input to ensure accuracy and provide opportunities for clarification and further elaboration.

### Data analysis

The data analysis was guided by Braun and Clarke’s six steps in reflexive thematic analysis [24] and Microsoft Word and Excel ® were utilized to manage the transcriptions, coding process, and development of potential themes.

The interviews in the two projects were digitally recorded and then verbatim transcribed. The interviews of the *improve-it* project and some excerpts from the *MSRHR project* were translated to English by a research assistant who is a native Arabic speaker. The first author then verified the accuracy and quality of the transcriptions and translations while familiarizing himself with the material by reading and listening to the interview.

The first and last author read the first two interviews and coded them separately and then discussed the coding procedure and preliminary patterns. The rest of the interviews were coded by the first author. All the authors deliberated on the codes and generated candidate themes. The first author wrote descriptions and arguments for the candidate themes, fostering ongoing discussion, negotiation, and refinement within the research team. This abductive approach allowed for iterative exploration between the initial codes and the evolving themes.

Finally, quotes were selected to illustrate and present the finalized themes in the manuscript. While most of the codes operated at a semantic level and the analysis followed an inductive experiential approach, efforts were made to identify latent meanings in the empirical data when applicable.

### Ethics approval and consent to participate

Before the interviews, we contacted the potential participants and provided them with both written and verbal details about the study, including the purpose of recording the interviews, the procedures for handling the data, and the voluntary and confidential nature of the study. When the interviews were conducted, we obtained oral consents and documented that in separate audio files. We were granted ethical approval for the study from The Swedish Ethical Review Authority under reference number 2020–05710 (Improve-it) and 2020–02816 (MSRHR).

### Methodological considerations

A major strength of this study is that it reports from Arabic speaking men’s lived experiences of family planning and decision making in a new setting rarely included in research.

Individual interviews were appropriate to foster open and comfortable conversation. The authors have various cultural understandings and come from different cultural and social backgrounds which bring diverse perspectives to the study. The first author is a Syrian-born Arabic-speaking man which facilitated trust of the participants. The first author acknowledges that these identities of being Arabic, man and migrant bestow upon him a complex blend of privileges and vulnerabilities that could have affected his understanding to the topic of this research but have also helped him reflect on his positionality with the team. The team has made consistent efforts to reflect on their positions and potential biases by engaging in a dialogue to consciously address the influence of personal experiences and knowledge on the research process.

Our sample size is in line with the recommendations of qualitative studies. The audit trail is described in the method section and we present quotes to illustrate the relation between the transcripts and the findings, in order to strengthen the trustworthiness [25]. However, it’s important to acknowledge that grouping all Arabic-speaking men together in one study can risk oversimplification and stereotyping. These individuals represent a diverse population, spanning various cultural, educational, and socioeconomic backgrounds which was apparent in our analysis. Our findings acknowledge the need to address the heterogeneity and the diversity of Arabic-speaking migrants in Sweden.

Additionally, the interviews were conducted online, and some were relatively short which might be because of the sensitive nature of the topic. This may have influenced the dynamics of the conversations and the depth of responses, potentially affecting the richness and nuance of the data collected. To maintain confidentiality, the interviews were recorded using a separate external dictaphone.

Although the participants are born in various Arabic countries, have different age and parental status, it is important to acknowledge that our participants’ perspective might not cover all Arabic-speaking migrants’ perspectives but exhibit a bias toward those with greater interest in the topic, higher education, and liberal perspectives.

The integration of data from two projects offers an enriched perspective on family planning among Arabic-speaking migrants in Sweden, however, this approach carries potential challenges including differences in aim and characteristics, for example, their length of stay in Sweden, necessitating careful data synthetization.

## Results

**Fig 1:**
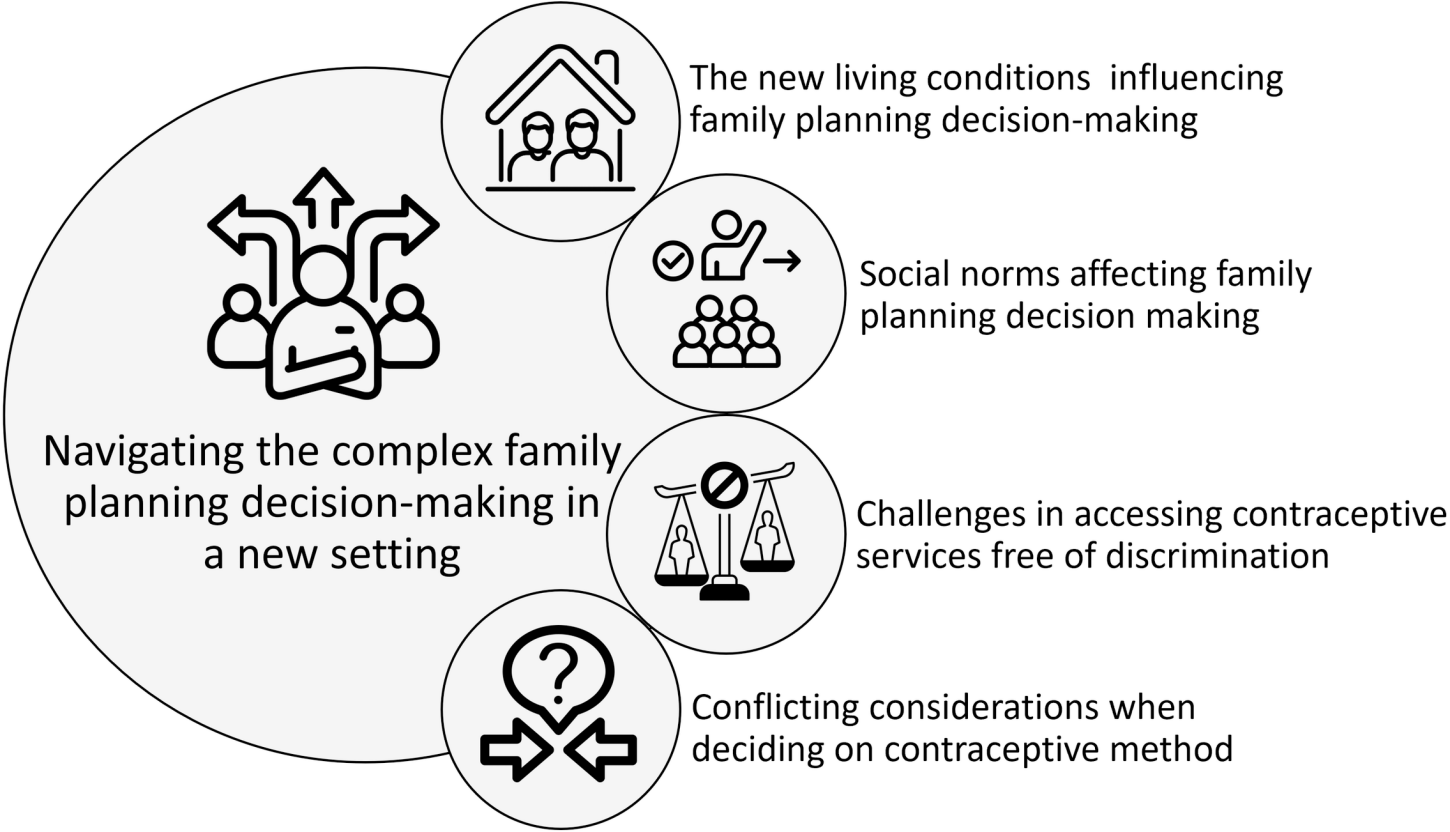
Navigating complex family planning decision-making in a new context; themes explaining Arabic-speaking men’s perspectives.

We developed an overarching theme “*Navigating the complex family planning decision-making in a new setting*”, based on four interconnected sub-themes (Fig 1). These sub-themes encompass *1) The new living conditions influencing family planning decisions*, *2) social norms affecting family planning decisions*, *3) challenges in accessing contraceptive services free of discrimination*, and *4) conflicting considerations when deciding on contraceptive method*. Each sub-theme brings forth a unique set of factors and perspectives that give deeper understanding of the multifaceted landscape and the diverse influences, challenges, and dynamics that shape individuals’ family planning decisions and inform strategies for promoting informed choices. Below are each of the sub-themes described.

### 1. The new living conditions influencing family planning decision-making

This sub-theme focuses on how men approach family planning and contraception based on their new living conditions in Sweden. The theme explains how the participants in the new context of Sweden consider factors like financial capability, childcare ability, personal development goals, and the impact of conflicts and political insecurities. The participants, however, embrace the uncertainties caused by their changing circumstances and priorities which affect their plans and decisions related to family planning.

The financial aspect played a crucial role in migrant men’s opinion about family planning decisions. Most participants referred to their financial (in)stability as migrants with new economic conditions when deciding on the number of children. High living costs, economic inflation, and strained financial situations were identified as influential factors that affect the number of children the participants wanted to have, i.e., limited the number of children.

“Yes, but it depends on our capabilities of course, I don’t want my children to be a burden on me or on the state, I don’t like that, […]. If I had the capacity to do my duty to these children, I will [have more children].” (Participant 4, improve-it project, 43 years, has children)

Besides the financial capability, the participants highlighted their ability to adequately care for children as something that very much affected their family planning decisions. Also, the need to work to meet the financial obligations made them less present for their children than they would like to be. Factors such as a mother’s health, the father’s involvement in raising children, and the desire for children to receive sufficient care and attention come into play. The men expressed challenges related to raising children in the absence of social support systems, both within the family and external networks, to assist in child-rearing responsibilities. The participants’ desire for their children to grow up within their familiar cultural frameworks makes them feel the need to take on greater child-rearing responsibilities in the new country, which make them more positive toward family planning.

“I mean, we raise our children within our Arabic tradition and education, so they can live and establish themselves in Swedish society but still preserve our religion, customs, and traditions. […] Considering that I am only here with my wife, so raising children is our responsibility, no parents, or relatives to help, here we have 100% responsibility for them, which make it more difficult.” (Participant 6, improve-it project, 35 years, has children)

The influence of instability and uncertainty on family planning choices provides insights into the resilience and adaptability of individuals in the face of challenging and changing circumstances. Most participants stated that family planning decisions are not static and may evolve over time. Embracing uncertainty meant that the participants may plan step-by-step, adapting their plans as circumstances change or as they reassess their situations and desires. Flexibility and the acknowledgment of the possibility for changing preferences underscore the dynamic nature of family planning choices.

“There is always a discussion about this subject, how many children we can have, we have almost determined that it is a maximum of 3, of course I don’t know, maybe we will change our mind in the future, I currently see that it is a maximum of 2” (Participant 5, improve-it project, 34 years, has children)

The aspirations for individual growth and development of the partners also impacted men’s opinion on family planning decisions. The participants indicated how establishing themselves in the new country and their own personal and professional goals align with their plans for having fewer children. Factors such as pursuing education, career advancement, and personal fulfillment often necessitate strategic decisions regarding the timing and spacing of children.

“Yes, for now, we only have one child because my wife is studying, I am studying, we cannot, I don’t think we have time to take care of another child.” (Participant 7, improve-it project, 31 years, has children)

Beside personal development, the participants referred also to maternal health and childcare ability as reasons for spacing. According to the participants, spacing children allows sufficient time for mothers to recover and maintain their health and helps avoiding the challenges of caring for two small children close in age which might leave them overwhelmed or feel “not enough”. Spacing also allows the older child to be more independent and understand the younger child’s needs.

“It is not about keeping a period between children, children will not be affected a lot […], also, I am not much affected…my wife will be affected the most, she is the one who will get pregnant and suffer the most in the beginning, that’s why she preferred to take a rest so my daughter would grow up a bit, to be able to depend on herself, to be able to go to the kindergarten” (Participant 5, improve-it project, 34 years, has children)

The participants indicated that their previous living conditions continued to shape their family planning decisions after arriving in Sweden. Experiences of war, political instability, and forced migration influenced how they thought about childbearing in a new context. Some participants explained that, coming from environments where child mortality was high due to conflict, having more children was seen as a way to ensure familial continuity and legacy. Others, however, expressed reluctance to expand their families due to the psychological toll of past trauma and a desire to protect their existing children. These deeply rooted experiences carried over into their lives in Sweden, informing how they approached reproductive decisions even in a more stable environment.

“In general, there were two sides, there were those who wanted to have many children because they were afraid of death and wanted to have children so that those children would take their names; and there were those who did not want to have children.” (Participant 1, improve-it project, 39 years, has children)

Diverse living conditions and personal preferences intertwine to shape family planning decisions. Recognizing the unique yet diverse living conditions and personal preferences of migrants can help support individuals in their family planning decisions.

### 2. Social norms affecting family planning decision-making

This sub-theme highlights the diversity of family planning decisions within Arabic-speaking communities, focusing on the individual differences that contribute to their complexity. It also addresses the perceptions of gender roles and the stereotype of the “controlling” Arab-speaking man.

Participants generally emphasized their willingness to share the responsibility of family planning with their partners yet in some instances noting male partners controlling behavior. Religious and social factors including the extended family members influence and clash with the couple’s plans and practices of family planning.

According to participants, couples are making collective decisions about the number of children they want, the use of contraceptive methods, and the spacing between children. The participants highlighted that mutual understanding and agreement on these matters are crucial for effective family planning. Both men and women are considered responsible for family planning, with an emphasis on active involvement of men and women in the decision-making process.

“Both [have responsibility] because the man is not having sex alone. It is a relationship between the two. It is the two. They both must have control.” (Participant 11, MSRHR project, 28 years, no children)

At the same time, the participants perceived women in most instances as the decision-makers in family planning especially in their agency and autonomy in choosing contraceptive methods. Many participants reported that their partners decided on the type of contraceptive methods they deem appropriate and made decisions after considering their own health and preferences. Rather than having ab authoritative role, the participants depicted their role as providing support and respecting their wives’ decisions and encouraging them to take the lead in this matter. They often described their role as being caring and supportive, both emotionally and financially, in matters related to family planning and being actively participating in child-rearing responsibilities.

“The body is hers and she is responsible for its safety. All I care about is her safety. Anything that causes harm to her health, I don’t do, and whatever she decides I support her 100%. Anything that makes her relax. The most important is her mental health.” (Participant 6, improve-it project, 35 years, has children)

However, some participants also highlighted instances of controlling behaviors exhibited by some Arabic-speaking men. These behaviors can stem from traditional gender roles, and patriarchal attitudes and lead to a disproportionate influence over family planning decisions.

“Now, it is personal. I don’t have any statistics, but there will always be such things, but I don’t know how common it is and to which degree. We come from a Middle Eastern society, unfortunately, a masculine and patriarchal society, so the female agency is affected by the society. I mean, there will certainly be cases where I would call it male suppression of the female by not letting her participate in such an issue [family planning], for example.” (Participant 10, MSRHR project, 35 years, has children)

The participants explained that there are differences in norms and values between and within the Arabic communities, individual differences. They emphasized that not all men or families have the same beliefs, attitudes, or preferences regarding family planning. Personal needs, education, beliefs, attitude, life situation, and familial norms all shape individuals’ perspectives on family planning and contraceptive methods. Therefore, they emphasized that it is crucial for healthcare providers to approach each case individually and consider the unique circumstances of the individuals involved to be able to tailor their support accordingly.

“Everyone is different, I don’t know much about Samir and Amira*, their situation, thinking, attitude, education, religion and their society, many things could make a difference. […] You can’t say that we are similar just because we are both Arab, we differ, according to the environment that surround us. I think you agree with me, and an Arabic person from Aleppo is different from another Arabic person from Aleppo, according to the environment.” (Participant 6, improve-it project, 35 years, has children) *personas used in the interview

Another factor that is related to social norms and might differ between individuals is religious beliefs. While some people might be discouraged by their beliefs to use contraception, the participants expressed personal opinions that differ from their beliefs. This clash between theory and practice is exemplified by some participants who adhere to moral values while making decisions that may contradict those values. Some participants expressed the fatalistic belief that the number of children is destined by God while still planning the use of contraceptives and for a desired number of children.

“It is right, I have a plan for this issue. Of course, it is destined by God, but we have a plan for this, Currently, we have one child, and we are planning for another one, and that is it, I wish.” (Participant 6, improve-it project, 35 years, has children)

Hence, decision-making processes are complex, and the individuals navigate and reconcile conflicting influences. Another complexity that the participants went through is the social norms related to the number of children. Social norms can directly shape individuals’ perceptions of the ideal number of children to have and can indirectly affect their decisions through the desires and opinions of extended family members. The participants, however, challenged the desires and opinions of extended family members emphasizing the couple’s autonomy and described a lower influence of extended family members on their family planning decisions in Sweden.

“Well, we did not let it play a large role, it didn’t really change our mind, we wanted to decide for ourselves, but the pressure of the parents is still present that we should have a child now. […] Of course, one of the reasons that helped us to achieve independence is being here in Sweden, I think if we were in mother country there will be more pressure from our parents.” (Participant 5, improve-it project, 34 years, has children)

Overall, this theme highlights the complexity of gender dynamics and social norms in relation to decision-making in family planning and underscores the importance of recognizing the diversity and individual differences among Arabic-speaking populations in Sweden.

### 3. Challenges in accessing contraceptive services free of discrimination

This theme focuses on the critical role that access to information and services play in individuals’ decision-making regarding family planning and contraception. Although most of the participants demonstrated familiarity and awareness about the importance of child-spacing and certain contraceptive options, they stressed the need for more detailed information about all contraception options including the pros and cons of various methods.

Despite the familiarity of some contraceptive methods, the participants exhibited, the participants perceived that they have limited knowledge about other contraceptive methods and expressed a need for more information and education on family planning to facilitate men’s engagement in reproductive health. More information would help Arabic-speaking men to get more involved in reproductive health and better support their partners in their decisions.

“I mean, in my opinion, if in terms of giving birth or children, or any matters related to family planning, the man should have a similar role as the woman. The same importance. He is not the one who will live the experience with pregnancy, but at least he should have more knowledge on the subject.” (Participant 19, 33 years, no children)

Although, the participants acknowledged various sources of information about family planning such as schools, family, friends, and social media, they noted that there are “few reliable Arabic websites” to access information. Therefore, there is a need for healthcare to offer comprehensive information about all contraceptive options. The participants discussed that midwives should address all concerns about side effects and provide translated materials in individuals’ native languages.

Although some participants thought that such needed information should be targeting their partners, other participants advocated that men should also be invited to discuss contraception together with their partners and should receive comprehensive information, for example, participant 1 wanted the midwife to:

“Explain the methods of contraception, because it is possible that there are people who want to try other methods…other than the IUD…I think that IUD is the most common used method” (Participant 1, improve-it project, 39 years, has children)

The participants felt that there are limited healthcare visits dedicated to discussing contraceptive methods. Some participants stated that they/their partner were the ones who initiated postpartum appointments for contraception. In many cases, these postpartum visits primarily focused on the requested method without exploring other options. According to the participants, the midwives did not usually discuss other contraceptive options rather only the ones the participants wanted to use, which is a lost opportunity to make informed decisions about family planning.

“We were in the health center and the talk was brief […] she showed me the pictures [showing contraception methods], she showed me a picture written in English, but she did not explain it.” (Participant 1, improve-it project, 39 years, has children)

Despite these limitations, the participants generally expressed positive experiences and satisfaction with contraceptive services and healthcare providers in Sweden. They appreciated the support received during consultations. Satisfaction with healthcare services extended beyond family planning, with participants expressing contentment with the medical help received for their children in Sweden.

“our experience was very good, there was no problem, after our first child was born they met us, only one meeting was made as I remember, this meeting was about our plans to the future, are we planning to have more kids or you want to stop, if we want to stop, do we have an idea of the methods to use? She has even given us leaflets to read at home” (Participant 5, improve-it project, 34 years, has children)

Yet, instances of perceived discrimination emerged with some participants expressing concerns about discriminatory questions, assumptions, and prejudices related to country of origin, religion, or gender roles. For example, Participant 1 pointed out that if the midwife is asking only women from the Middle East if they are forced to use or remove an IUD, is exhibiting stereotypes and discriminating against migrants with Middle Eastern backgrounds:

“if the question is directed only to us because we are migrants or have Middle Eastern background, then the question is provocative. Honesty, we are in Sweden in the 21st century with the social media and the Internet. I do not think that there is a woman whose husband forces her to do anything.” (Participant 1, improve-it project, 39 years, has children)

Therefore, the participants emphasized the importance of impartiality, cultural understanding, and professionalism from healthcare providers. They emphasized the need for receptive communication without imposing personal ideologies, underlining the significance of unbiased, culturally sensitive, easily accessible information to empower informed family planning decisions.

“They [healthcare workers] should not plant their ideas, they must be very neutral and very trustworthy, they must be without agendas or ideologies, that they think they are right and want to enforce them on others, they must be neutral.” (Participant 2, improve-it project, 36 years, has children)

Overall, the theme underscores the importance of providing comprehensive, culturally sensitive, and easily accessible information to empower individuals and families in making informed decisions about family planning.

### 4. Conflicting considerations when deciding on contraceptive method

This sub-theme presents factors that played a significant role in shaping men’s opinions when deciding a contraceptive method including familiarity with the methods, their safety, effectiveness, and convenience, the fear of side-effects, and the taboos surrounding the use of some methods.

Most participants demonstrated familiarity with some contraceptive methods based on previous experiences of using, for example, IUDs, contraceptive pills, and condoms and they shared positive experiences with these methods, highlighting their effectiveness in preventing pregnancy. However, especially in relation to the side effects of using some contraceptives on women’s health, some participants preferred withdrawal and other natural methods, indicating familiarity with a range of contraceptive choices among the participants. The familiarity with specific contraceptive methods depended on the pattern of contraception methods people use in the participants’ countries of origin. The participants highlighted also that their patterns of contraceptive use did not change after migrating to Sweden.

“Our people also used it, I mean, I don’t know if the hormonal method is good or not, it could be good, but I have no background about it, honestly. People tend to the things they are more familiar with, especially in matters related to health and these sensitive matters such as childbearing.” (Participant 1, 39 years, has children)

On the other hand, taboos and conservative attitudes towards sexuality hindered open discussion about contraception in the society particularly between men influencing their contraceptive knowledge and choices. These taboos, for example, are observed in the context of condom use, where social norms affect the acceptability of using condoms within marital relationships. However individual differences are highlighted with some participants describing using condoms as a contraceptive method of choice or even stating condoms should be the first choice which highlight the difference among Arabic-speaking communities and individuals.

“If you don’t want to get pregnant, you should use condoms. You should not use things that enter your body and play with your hormones or something. This is my opinion for both I mean because everything that play with hormones, honestly, I don’t know, for me, it is not necessary.” (Participant 12, 24 years, no children)

Nevertheless, most of the participants indicated that women’s health is the number one priority when it comes to choosing contraceptive methods. Safety concerns and fears of potential side effects associated with some contraceptive methods significantly influenced their opinion to use or avoid specific contraceptive methods, specifically hormonal methods. These concerns were due to both legitimate potential side effects and/or fear of unreported side effects, namely infertility.

“I think it does not affect the man but the woman, according to what I have read, it might cause hormonal changes, problems with the menstrual cycle, fatigue, and ovarian cyst” (Participant 7, 31 years, has children)

The participants balanced considerations of convenience and effectiveness against safety concerns which was influenced by their experiences and familiarity of some methods. The uncertainties and instability we referred to in the first theme led some participants to prioritize methods that are more effective in preventing unintended pregnancies and those that fit seamlessly into their everyday “stressed” lives. Factors such as convenience, ease of use, and the ability to maintain a normal routine were significant considerations when selecting a contraceptive method.

“no one recommended anything other than an IUD…and you live your life normally…that was the word “live your life normally” …you don’t have to use condoms and you don’t have to take pills, and you forgot to take your pills and you didn’t […] you put your mind at ease, because in this country, you are stressed and under a huge pressure” (Participant 3, 39 years, has children)

Understanding the role of these concerns, the fear of side effects and the hinder to an open discussion about family planning among men, highlight the importance and need of information and family planning services.

## Discussion

In this study, we explore various aspects of family planning among Arabic-speaking migrant men in Sweden, highlighting the complexity of navigating the processes of family planning decision making within a new setting. Multiple factors influenced these decisions, including altered living conditions, gender dynamics, and social norms within Arabic-speaking communities, along with the diverse considerations when choosing contraception methods, and challenges in accessing comprehensive information and contraceptive services.

Our findings illustrate how Arabic-speaking men were embracing changes in life circumstances. Most men reported these changes as reasons of not having definite plans. One reason could be that the dramatic change in their lives due to forced migration made them realize that changing and reconsidering their plans are inevitable. These experiences of changed life circumstances impact family planning, emphasis needs of building trust and nuanced preunderstandings among HCPs also in SRH services as described in other contexts [26].

Arabs and Muslims are frequently represented in Western media and public discussions as violent, untrustworthy, and a threat to Western values [27,28]. The Swedish National Council for Crime Prevention (Brå) emphasizes in its report “Islamofobiska hatbrott 2021:3” that Muslims in Sweden often face bias and suspicion when interacting with authorities. Such assumptions if held by healthcare providers can influence healthcare quality. The diversity among Arabic-speaking couples highlights the importance of understanding and respecting the differences in order to tailor support and healthcare services effectively. It shows needs for sharing different perspectives of Arabic-speaking men’s roles in family planning, emphasizing that there is no one-size-fits-all approach. Taken together, our findings strengthen needs of engaging Arabic speaking men in contraceptive conversations and educational initiative. Previous research underscores the importance of engaging both women and men since it is thought to encourage communication between couples and ease women’s ability to make life choices in family planning and to access contraception [29,30].

The study highlights the need for men to have access to comprehensive information about contraceptive options, a need that has also been described in other studies [12,30]. Comprehensive information is crucial for making informed decisions about family planning and enhancing satisfaction with family planning services. However, some scholars argue that the prevailing ideology of gender equality and the implicit norms among healthcare workers in Sweden could potentially limit their ability to diversify their approach to contraceptive counseling which could hinder the engagement of male partners and subsequently restrict women’s access to appropriate support [31]. Engaging men in counselling might directly address their concerns or misconceptions about contraceptive methods that hinder their own use and that of their partner.

Our findings stress future needs for designing services by including the components of building trust, quality, access and follow-up support as in the person-centered framework for contraceptive care [32]. This is likely relevant when improving family planning services among migrants, since choice of contraceptive methods is a sensitive decision for many both women [33,34] and men [30] worldwide. Need of support and building trust should be considered when developing systems aimed to facilitate dialogues between migrant men and health care providers in SRH services since family planning decision-making in a new setting is complex. Future health care and research should focus on how to co-design systems for family planning services together with both women and men with different cultural background to improve quality and safety [35,36].

## Conclusions

This study underscores Arabic-speaking men’s willingness to be active partners in family planning and contraceptive decision-making. It emphasizes various perspectives that need to be acknowledged such as living conditions in a new context, and social norms affecting family planning and the process of contraceptive decision making.

The findings emphasize the need for improved, non-discriminatory contraceptive services and more effective information dissemination. Additionally, the study highlights the diversity of individual decision-making and the necessity of impartial and inclusive family planning for all women and men in Sweden. Engaging men in family planning empowers individuals and families to make informed choices that align with their unique circumstances and aspirations.

## Supporting information

S1 File Interview guide for the Improve-it project. (DOCX)
