# Supplementary material for: Navigating complex decision in a new setting; perspectives of Arabic-speaking migrant men in Sweden on family planning decision making
Source: PLoS One. 2025 Jun 11;20(6):e0325935. doi: 10.1371/journal.pone.0325935 (PMC12157100; doi:10.1371/journal.pone.0325935)
Supplement: S1 File — (DOCX) [file pone.0325935.s001.docx]

# **Interview guide**

**Aim of the study**

To explore migrant men's perceptions of planning pregnancies, decision-making and experiences of talking about family planning and contraceptive methods in general and in particular in connection with pregnancy and delivery.

**Topics:**

1. Pregnancy planning in relation to family planning and contraceptive methods.

2. knowledge/use of contraceptives.

3. Men's involvement in family planning/contraceptive methods.

4. Experience with family planning/contraceptive counseling in Sweden and desired information and approach to postpartum counseling (where, when, how, by whom).

**Advice to the moderator:**

- Mirror their words i.e. repeat the words they choose to use.

- You also mirror to get confirmation that you have understood the information correctly.

- It is important that the moderator remains neutral and does not share their own opinions.

**Follow-up questions to ask:**

- Can you tell me more about?

- Can you explain it to me?

- Can you give examples?

- What do others say/What do you think?

- Do others recognize themselves or what are your experiences?

- Have you heard about someone else's experiences?

- Continuously ask: "what do others think about x,y,z?".

**Interview guide for men's focus groups (IMPROVE it)**

1. Start with: Today we are going to talk about having children and family planning. We will start with your perceptions of planning for pregnancy and contraceptive use and your role in these, and then we will talk more about your experience of talking about contraceptive methods in Sweden. But first, what does it look like for you, is family planning something you usually talk about with family and friends?

**Theme 1: - Pregnancy planning in relation to family planning and contraceptive methods.**

1. Many families in Sweden plan/decide how many children they want to have - what are your thoughts on this?

-How do you think about family planning/contraceptive methods?

-How do you think about the number of children? Pregnancy intervals?

-How do you decide how many children to have?

-How do you/your partner talk about how many children you will have?

-What is important to you?

**Theme 2: knowledge/use of contraception.**

1. Which contraceptive method do you know?

The moderator then shows a picture of different contraceptive methods:


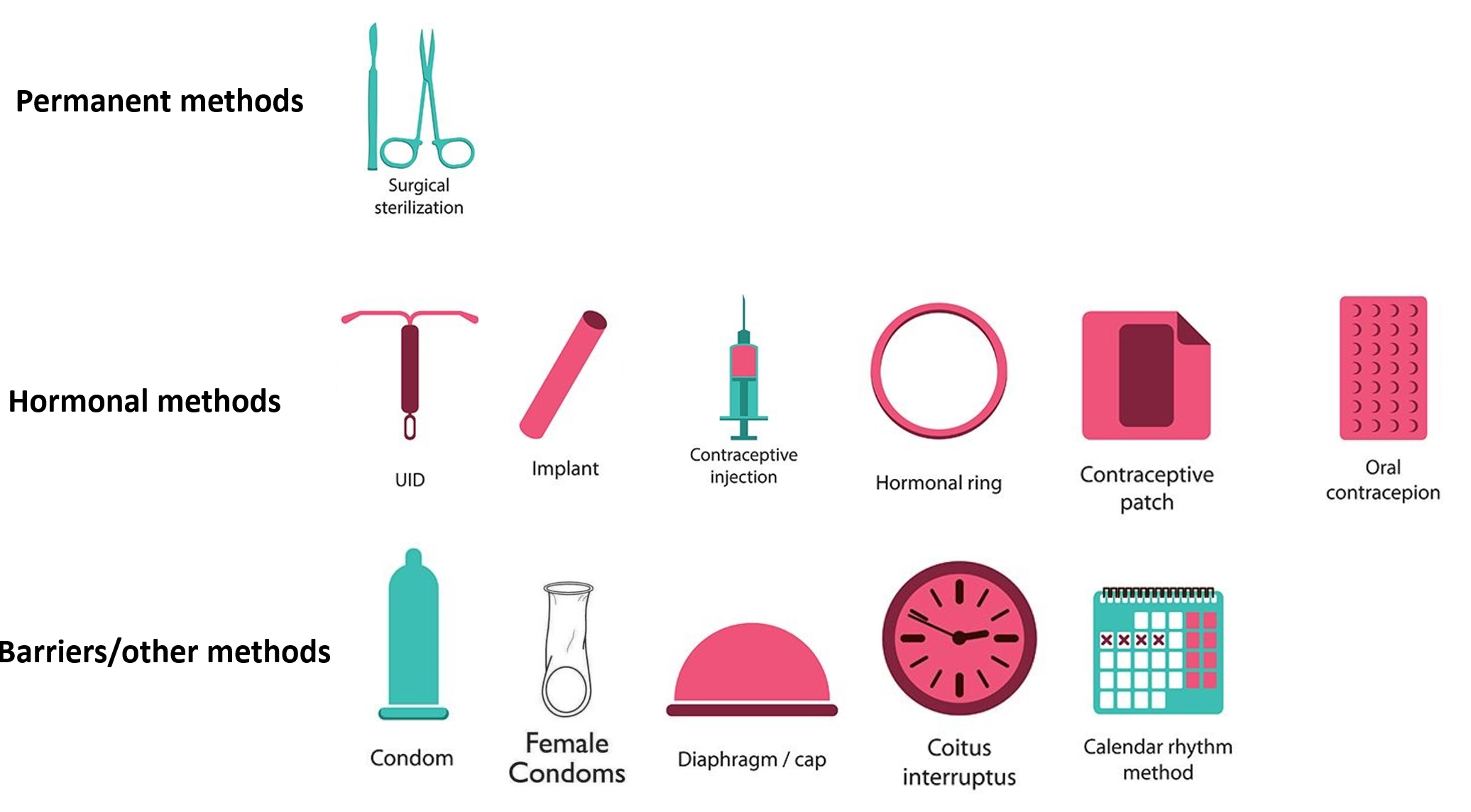


1. How do you view the use of contraceptive methods? Or what do you think about contraceptive methods?

-How do men in general view contraceptive use? For/against? Why?

- Some couples use contraceptive methods and some do not. What do you think about the reason for this? What is the reason for using some methods but not others?

-using contraception after the baby is born/expected.

-Good/bad for women's bodies/health.

-What influences your decision to use contraceptive methods?

-what is good or bad about contraceptive methods?

-What is your experience with contraceptive methods?

-How did you get knowledge/information about contraceptive methods?

-From where have you received knowledge/information about contraceptive methods (home country, Sweden)?

**Theme 3: Men's involvement in family planning/contraceptive methods.**

1. How do you see your role as a partner in decisions about contraceptive methods? Which other people influence you/your partner/the joint decision on contraceptive use or which methods you use?

**Theme 4: Experience with family planning/contraceptive counseling in Sweden and desired information and approach to postpartum counseling (where, when, how, by whom).**

1. What is your experience of getting advice on contraceptive methods in Sweden?

-Was your partner invited to talk about contraceptive methods after childbirth? (Did she/you go? Why? Why not?)?

-Were you present during this or other contraceptive discussions with your partner? (Why? Why not?)

-How was your experience of being with your partner in conversations about contraceptive methods? and during pregnancy/postpartum?

-Have you heard from anyone else who has different experiences?

-Where and from whom would you like to receive information on contraceptive methods?

-Have you lacked anything when you received information about contraceptive methods? what?

-digital counseling

***A case (Moderator read/show picture):***

**Samira and Amir come 10 weeks after delivery. They have a small child. They do not know if they want to have more children or when.**

when it comes to deciding/planning pregnancy and using contraceptive methods:

-What do you think the provider should help them with (making appointments, asking questions, inserting contraceptives...)?

What information about contraception should the provider give/bring up at this visit?

-Is there anything to avoid talking about? What is it? Why?

-What should the midwife know about families that have recently arrived in Sweden? (impact of the family, role of mother, role of men...)

- How do you want to be invited to/during conversations about contraceptive methods?

*What are the possible developments?*

*Is there anything else you think is important...?*

1. What do you think about talking about/deciding on contraception when your partners are still pregnant?

-timing?

**To the moderator: In case it doesn't come up:**

Here's what some men say:

*"Contraceptive methods are good for giving women a rest before the next pregnancy, but contraceptive methods are bad for women's bodies."*

-What do you think about that? -What has been your experience?

*"Men are afraid that contraceptive methods prevent women from having more children."*

-What do you think about that? -What has been your experience?

*"Men are not allowed to be present when midwives talk to women about contraception."*

-What do you think about that?

*"Men want to be involved in decisions about contraception."*

-What do you think about that?

**Closing questions:**

Now we have talked about your experiences of discussing contraceptive methods with midwives, is there anything you would like to add that we haven't talked about or that no one has mentioned?

We are developing counseling for migrant women and men together with midwives. Are there any of you who would like to be involved and who we can contact?

Write down your contact details: name, phone number, email.

**Note to the moderator/observer:**

After the focus group, participants should fill in background data.

If there is a need for contraception - offer/guide how and where to get advice.
